# Retraction: Blood Parameter Profiles and Their Clinical Implications in Hypertensive Patients: A Retrospective Chart Review

**DOI:** 10.7759/cureus.r164

**Published:** 2025-01-24

**Authors:** Ahmed S Al Zomia, Zia Sabah, Mosab Deajim, Abdullah H Alamri, Ghufran B Asiri, Lama A Lahiq, Wajd Alhadi, Nasser A Alwaqdi

**Affiliations:** 1 College of Medicine, King Khalid University, Abha, SAU; 2 Department of Medicine, King Khalid University, Abha, SAU; 3 Department of Medicine and Surgery, King Khalid University, Abha, SAU

This article has been retracted by the Editors-in-Chief due to extensive errors that cast doubt on the validity of the work. These errors include unrealistic patient lab values that are incompatible with life, methodological errors, a lack of critical analysis, and contradictions in the data and inclusion/exclusion criteria. The significant number of errors necessitates retraction rather than the issuance of a correction. As a result, this article has been retracted. The authors disagree with the journal's decision to retract.

